# Restricted language access during childhood affects adult brain structure in selective language regions

**DOI:** 10.1073/pnas.2215423120

**Published:** 2023-02-06

**Authors:** Qi Cheng, Austin Roth, Eric Halgren, Denise Klein, Jen-Kai Chen, Rachel I. Mayberry

**Affiliations:** ^a^Department of Linguistics, University of Washington, Seattle, WA 98195; ^b^Department of Linguistics, University of California San Diego, San Diego, CA 92093; ^c^Department of Radiology, University of California San Diego, San Diego, CA 92093; ^d^Department of Neuroscience, University of California San Diego, San Diego, CA 92093; ^e^Neurology and Neurosurgery, Montreal Neurological Institute, McGill University, Montreal H3A 2B4 Canada

**Keywords:** deafness, sign language, brain development, surface-based analysis, language deprivation

## Abstract

Due to the ubiquitous nature of language in the environment of infants, how it affects the anatomical structure of the brain language system over the lifespan is not well understood. In this study, we investigated the effects of early language experience on the adult brain by examining anatomical features of individuals born deaf with typical or restricted language experience in early childhood. Twenty-two deaf adults whose primary language was American Sign Language and were first immersed in it at ages ranging from birth to 14 y participated. The control group was 21 hearing non-signers. We acquired T1-weighted magnetic resonance images and used FreeSurfer [B. Fischl, *Neuroimage* **62**, 774–781(2012)] to reconstruct the brain surface. Using an a priori regions of interest (ROI) approach, we identified 17 language and 19 somatomotor ROIs in each hemisphere from the Human Connectome Project parcellation map [M. F. Glasser *et al.*, *Nature* **536**, 171–178 (2016)]. Restricted language experience in early childhood was associated with negative changes in adjusted grey matter volume and/or cortical thickness in bilateral fronto-temporal regions. No evidence of anatomical differences was observed in any of these regions when deaf signers with infant sign language experience were compared with hearing speakers with infant spoken language experience, showing that the effects of early language experience on the brain language system are supramodal.

## Significance

The current study provides direct evidence of the critical role of early language experience on the development of language-relevant regions in the brain. The results show selective effects in inferior frontal and posterior temporal regions, as well as bilateral effects, showing that the brain language network is sensitive to language experience during early childhood. The results extend previous studies showing that language experience during early life is necessary for the brain language system to fully emerge.

Language is a uniquely human cognitive ability involving a specialized and distributed left-lateralized fronto-temporal brain network (1–3). To date, however, how this highly efficient and complex language network emerges in the brain during early human development is poorly understood. Much of postnatal brain development is experience dependent. A series of neural processes, including synaptogenesis, pruning, and myelination, are shaped by environmental experience and learning (4–6). Language-related regions (including inferior frontal gyrus (IFG), temporal regions, and inferior parietal regions) grow more slowly compared with sensory-motor regions (7–9), suggesting an extended neural plastic period when language from the environment can shape this brain network.

Previous studies have found that the variation in the amount of language children experience affects the rate at which they develop language (10–15). Parental vocabulary diversity and syntactic complexity predict advances in children’s language development and the effect increases with age (N = 1,058, r = 0.35) (16). Randomized clinical trials and parent coaching intervention studies have found reliable increases in children’s expressive language skills with increases in parent–child interactions (17–21). Indeed, the more conversational turns 4-to-6-y olds participated in, the greater the BOLD signal changes they showed in brain language areas while performing language tasks. The effects included greater white matter connectivity in the left hemisphere (LH), arcuate and superior longitudinal fasciculi (22, 23) and increased cortical thickness in the LH IFG and supramarginal gyri (24). Five-to-9-year-olds who engaged in more parental conversations showed increased cortical area in the LH perisylvian regions (25).

Thus, variation in the amount of language children experience is associated with gray and white matter development in the brain language system. Here we ask whether restricted language in the child’s environment shows effects on measures of cortical structure in the adult brain.

Deafness often severely restricts the number of conversations infants and young children can perceive and participate in, because they cannot hear spoken language, and lipreading is too impoverished a language signal to enable spontaneous language acquisition. Although some deaf children successfully acquire spoken language with intensive intervention, many do not. Sign language is visually accessible to children born deaf, and is acquired spontaneously when present in the environment, but it is absent from the environment of most deaf children. Examining the brain developmental outcomes of varying childhood language experience in this population can shed light on the role of early language experience on the emergence of the language network and provide insights on how to better support deaf children’s language and brain development.

Behavioral studies have repeatedly found a negative relation between the age-onset of immersion in a sign language environment and the outcome of language learning in the population of deaf signers (26–32) (see ref. 33 for a review). Neurolinguistic studies of the phenomenon have found reduced activation in classic language regions or atypical activation patterns (34–36). In particular, Mayberry et al. (34) found a negative correlation between age of initial sign language immersion (between 0 to 14 y old) and BOLD activation in several left fronto-temporal language regions using sentence processing tasks in American Sign Language (ASL).

Few studies have examined the effects of restricted language experience during childhood on the anatomical structure of the brain language system. Here we analyze the dataset from Pénicaud et al. (37) using a surface-based morphometry (SBM) analysis (38) to focus on both language and non-language regions of interest (ROIs) derived from a multi-modal cortical parcellation map (39). The previous whole-brain voxel-based study found that restricted language experience in childhood was negatively correlated with grey matter in occipital areas, with no effects observed in language-relevant regions. Here we reanalyze the data set with SBM to answer three questions. First, can surface-based analysis reveal possible effects of restricted language experience on anatomical structures in the adult brain? If so, we should observe age of exposure effects on the language ROIs but not on non-language ROIs. Second, if structural effects are observed, the question is whether all language-relevant regions are equally affected, or alternatively whether some regions are more affected than others. Third, are the effects of early language experience on the development of the cortical areas of the brain language system altered by the sensory-motor modality of the early language? We answer the last question by comparing surface-based analyses of the participants in the present study, deaf signers who experienced ASL from birth, with hearing non-signing speakers from previous studies at the Montreal Neurological Institute (MNI) matched by handedness and age.

## Results

### Effects of Early Language Experience.

**Fig. 1.**
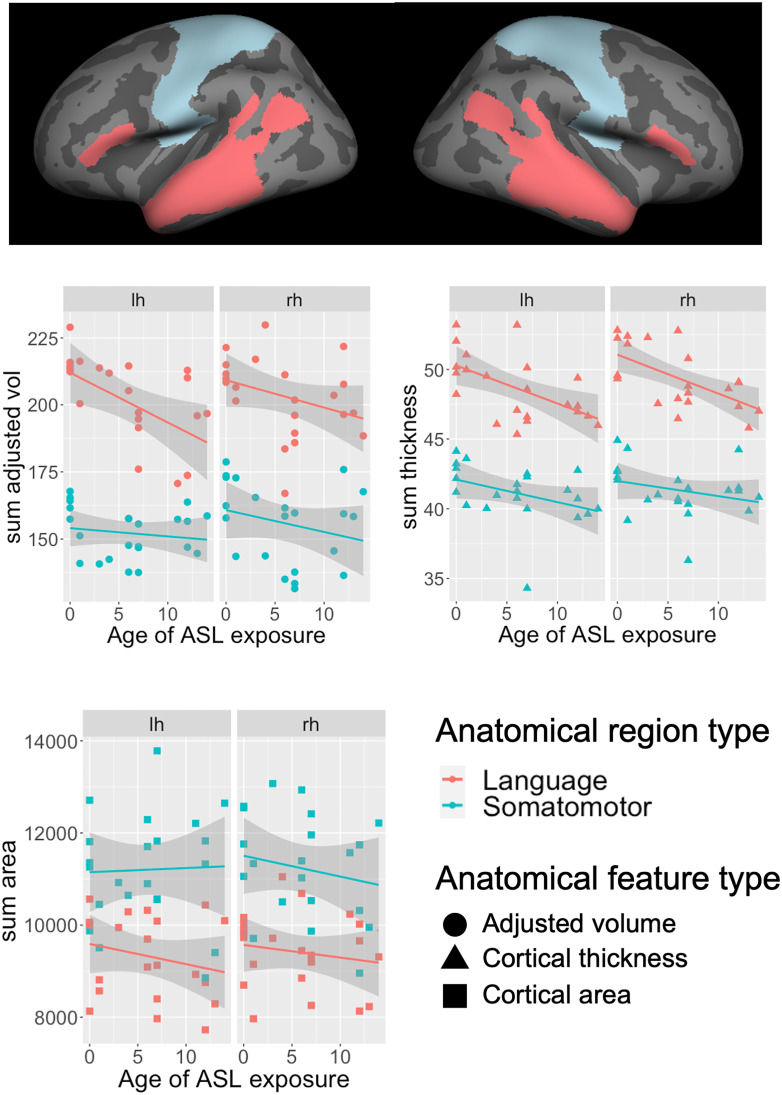
Age of ASL exposure (AOA) effects on three cortical features (adjusted volume, cortical thickness, cortical area) in bilateral language (red) and somatomotor (blue) brain regions selected from the HCP cortical parcellation map.

To investigate the first question, we examined the effects of three variables, Age of ASL Acquisition (AOA), type of brain regions (language vs. somatomotor), and hemisphere (LH, RH) using three cortical measures: adjusted volume, cortical thickness, and cortical area. We pre-selected ROIs in each hemisphere, 17 in target language areas, and 19 in somatomotor areas as controls. The participants were all born deaf but varied in their age of initial daily experience with ASL (see Table 2 under *Method* for participants’ details). Fig. 1 shows the effect of AOA on summed adjusted volume (Fig. 1*A*), summed cortical thickness (Fig. 1*B*), and summed cortical area (Fig. 1*C*) in language vs. somatomotor regions.

**Table 2.** Background of the deaf participants

ID	ASL AOA	Group	Age^*^	Gender	School entry age^*^	Type school^†^	d’GJ^‡^	d’HJ^‡^	PC^§^	PA^§^
01	0	Native	53	f	5	Residential	2.59	3.57	12	11
02	0	Native	26	f	3	Oral	1.93	1.65	11	13
03	0	Native	28	f	3	TC	1.93	1.65	11	15
04	0	Native	25	f	3	TC	3.25	1.78	7	7
05	0	Native	33	m	4	TC	2.21	2.84	14	15
06	1	Native	31	f	NA	NA	2.03	2.62	10	16
07	1	Native	26	f	3	Mainstream	2.07	0.91	NA	NA
08	3	Native	34	m	3	Oral	1.05	2.49	10	11
09	4	Early	36	m	4	TC	3.25	1.78	11	15
10	6	Early	35	m	4	Oral	1.93	1.81	12	15
11	6	Early	52	m	5	Oral	0.71	2.62	11	11
12	6	Early	35	m	3	Oral	0.39	2.16	12	12
13	7	Early	60	f	7	TC	1.46	0.73	11	9
14	7	Early	53	m	7	Oral	0.6	2.38	NA	NA
15	7	Early	54	f	7	Oral	1.7	2.14	NA	NA
16	7	Early	27	f	2	Oral	2.03	2.62	NA	NA
17	11	Late	29	m	5	Oral	1.25	0.17	10	8
18	12	Late	37	m	3	Oral	0.59	2.26	8	13
19	12	Late	31	f	3	Oral	2.07	3.23	10	15
20	12	Late	57	m	6	Oral	0.16	1.14	NA	NA
21	13	Late	34	f	3	Oral	1.11	1.3	10	7
22	14	Late	53	m	3	Oral	0.01	1.18	9	11

^*^AOA × Age: R^2^ = 0.129, t = 1.72, *P* = 0.101; AOA × Years of ASL experience (Age minus AOA): R^2^ = 0.003, t = −0.25, *P* = 0.804; AOA × Age of school entry: R^2^ = 0.014, t = 0.52, *P* = 0.610.

^†^Residential = dorm living with deaf signing peers with some classrooms TC and some classrooms Oral; TC = Total Communication (simultaneous speech & sign); Oral = speech only with sign and gesture actively discouraged; Mainstream = neighborhood school; NA = not available.

^‡^d’ for ASL Grammatical Judgment scanner task performance × AOA: R^2^ = 0.416, t = −3.17, *P* = 0.001; d’ for Phonemic Hand Judgment scanner task performance × AOA: R^2^ = 0.078,t = −1.30, *P* = 0.207.

^§^Scaled score for Picture Completion, PC × AOA: R^2^ = 0.119, t = −1.42, *P* = 0.176; Scaled score for Picture Arrangement, PA × AOA: R^2^ = 0.091, t = −1.22, *P* = 0.0240.

Using age and gender as covariates and ROI as random errors in a linear mixed model, with LH and the somatomotor regions as baselines, we first analyzed the adjusted volume and found no significant AOA effect for the control somatomotor ROIs in the LH (β = −0.0085, se = 0.0247, t = −0.344, *P* = 0.73) but a significant interaction between AOA and the language regions (β = −0.092, se = 0.034, t = −2.675, *P* = 0.0075), suggesting a negative association between duration of restricted language experience in childhood and adjusted cortical volume only in the language-relevant regions in the LH. No hemisphere interactions with AOA and/or type of ROIs were found, suggesting undetectable laterality effects in the language regions. As for cortical thickness, we found a significant AOA effect for somatomotor ROIs (β = −0.0064, se = 0.0023, t = −2.73, *P* = 0.006) and a significant interaction between AOA and the language regions (β = −0.0071, se = 0.0032, t = −2.162, *P* = 0.03) in the LH, suggesting negative AOA effects in both somatomotor and language regions but to a greater extent in the language regions. Again, there were no hemisphere interactions with AOA and/or type of ROIs, suggesting similar AOA effects in bilateral language regions. A similar linear mixed model using cortical area as the dependent variable found no effects for AOA.

These results indicate that restricted access to language during childhood is negatively associated with cortical thickness and adjusted cortical volume in language-relevant regions in both hemispheres.

**Fig. 2.**
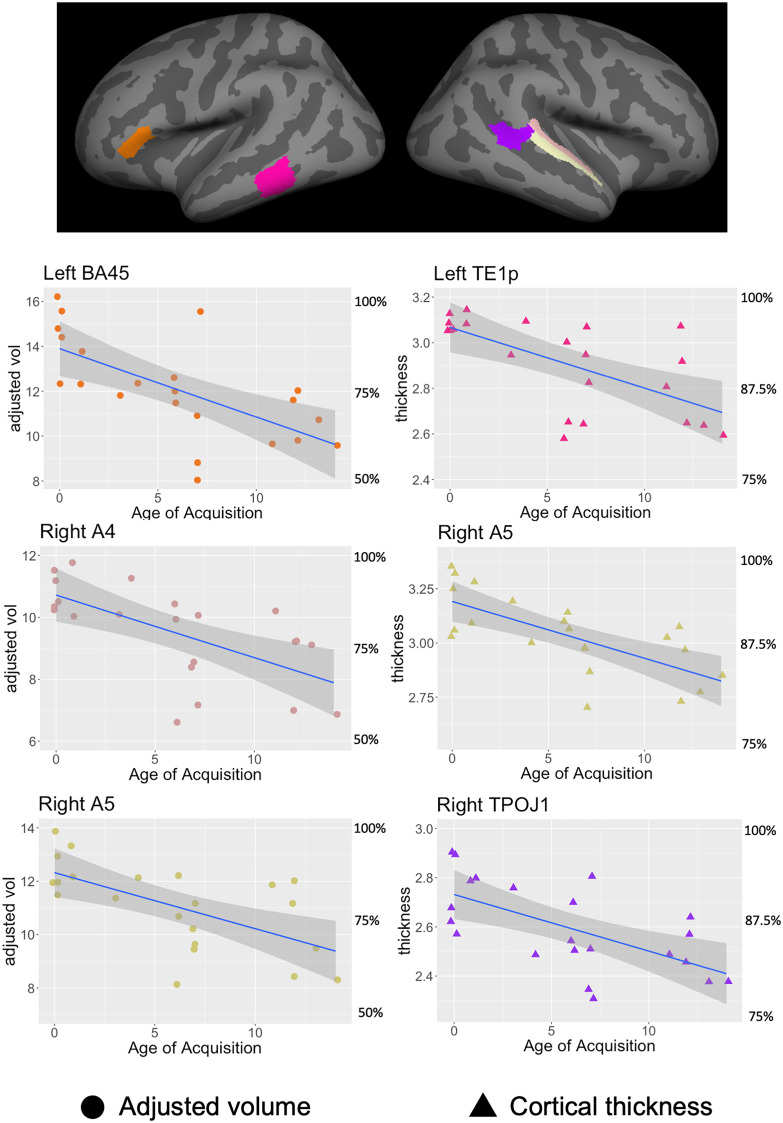
Correlation between Age of ASL exposure and selected cortical features that show highly significant effects (*P* < 0.01). BA45: brodman area 45 (IFG, pars triangularis); TE1p: area TE1 (middle temporal) posterior; A4: auditory complex 4, dorsal pSTG; A5: auditory complex 5, ventral pSTG; TPOJ1: temporo-parieto-occipital junction 1.

To further specify the nature of the bilateral AOA effects and answer the second question, we compared the altered anatomical features (adjusted volume and cortical thickness) for each selected language-relevant ROI across all deaf signer participants, using AOA as the independent variable. The cross-sample distribution for each anatomical measurement is given in *SI Appendix*, Tables S1–S3. To control for the family-wise error rate, we used the max-T permutation method to correct for correlated multiple comparisons (40) and identified two language brain regions in the LH and three language brain regions in the right hemisphere (RH) that showed a significant AOA effect (corrected *P* < 0.05). These regions are left BA45 (adjusted volume, β = −0.305, se = 0.077, t = −3.92, uncorrected *P* = 0.0008, corrected *P* = 0.024, adjusted R^2^ = 0.406); left TE1p (cortical thickness, β = −0.026, se = 0.006, t = −3.807, uncorrected *P* = 0.001, corrected *P* = 0.033, adjusted R^2^ = 0.391); right A4 (adjusted volume, β = −0.202, se = 0.054, t = −3.702, uncorrected *P* = 0.001, corrected *P* = 0.040, adjusted R^2^ = 0.376); right A5 (adjusted volume, β = −0.210 se = 0.057, t = −3.626, uncorrected *P* = 0.001, corrected *P* = 0.048, adjusted R^2^ = 0.366, cortical thickness, β = −0.026, se = 0.005, t = −4.429, uncorrected *P* = 0.0002, corrected *P* = 0.008, adjusted R^2^ = 0.469), and right TPOJ1 (cortical thickness, β = −0.022, se = 0.006, t = −3.63, uncorrected *P* = 0.001, corrected *P* = 0.048, adjusted R^2^ = 0.367). Fig. 2 shows the location of these regions as well as the correlations between AOA and the corresponding cortical measurements. Henceforth we will only discuss these corrected significant findings.

### No Evidence of Effects of Early Language Modality.

**Fig. 3.**
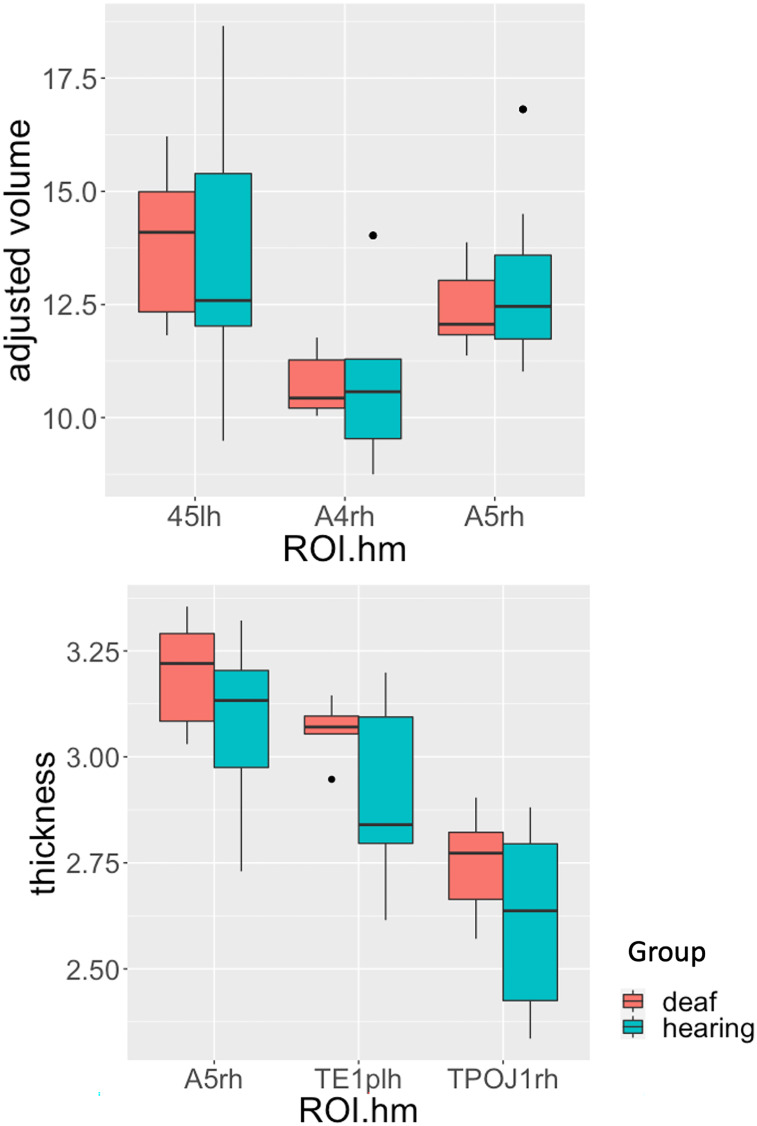
Hearing native speakers and deaf native signers group distribution for adjusted volume in left BA45, right A4, and right A5, and cortical thickness in left TE1p, right A5, and right TPOJ1. The box plot shows the distribution of each group. The top of the box shows the higher quartile (25%), the middle bar shows the median (50%), and the bottom of the box shows the lower quartile (75%). 45: brodman area 45 (IFG, pars triangularis); TE1p: area TE1 (middle temporal) posterior; A4: auditory complex 4, dorsal pSTG; A5: auditory complex 5, ventral pSTG; TPOJ1: temporo-parieto-occipital junction 1. Lh: left hemisphere; rh: right hemisphere.

Given that the duration of restricted language experience during childhood affects cortical measures in adults born deaf, the next question is whether the modality of the language experience of the deaf signers through the visual-motor modality affects the anatomical structure of the selected language regions of the brain. To investigate this question, we compared the deaf signers whose parents signed to them from birth (N = 8) with a group of hearing non-signers who experienced speech from birth (N = 21). Linear models, with group (hearing vs. deaf) as a main factor and age and gender as covariates, showed no significant sensory-modality effects associated with the infant language experience in all six brain anatomical features with corrected significant negative AOA effects. There was a nonsignificant trend (*P* < 0.08) for increased cortical thickness in the deaf native signers in right TPOJ1 (β = 0.156, se = 0.076, t = 2. 055, *P* = 0.050). Fig. 3 shows the group median with interquartile range distribution for the five brain regions/anatomical features with AOA effects (with corrected *P* < 0.05), where no evidence of differences was observed between the two groups with infant language experience: the deaf participants through sign language and the hearing participants through spoken language. (For cross-sample distribution for each anatomical measurement, see *SI Appendix*, Tables S1–S3).

Given the small sample size, we further examined the null results using Bayesian statistics. Table 1 shows the posterior distributions of deaf native anatomical features in all six anatomical features when compared with hearing individuals, with age and gender as controls.

**Table 1.** Posterior distribution of AOA-relevant anatomical features for deaf native signers as compared to hearing non-signing controls

Region	Measure	Median	95% CI	pd (%)	ROPE	% in ROPE	Test for practical equivalence
Left 45	Adjusted volume	−0.10	[−2.07, 1.85]	54.17	[−0.24, 0.24]	19.89	Undecided
Right A4	Adjusted volume	0.21	[−1.13, 1.41]	63.20	[−0.15, 0.15]	18.15	Undecided
Right A5	Adjusted volume	−0.56	[−2.10, 0.81]	77.40	[−0.17, 0.17]	14.13	Undecided
Left TE1p	Cortical thickness	0.12	[−0.02, 0.26]	95.80	[−0.02, 0.02]	4.68	Undecided
Right A5	Cortical thickness	0.13	[−0.02, 0.27]	95.55	[−0.02, 0.02]	4.05	Undecided
Right TPOJ1	Cortical thickness	0.16	[−0.01, 0.31]	96.80	[−0.02, 0.02]	2.16	Undecided

These findings largely accord with the findings using null hypothesis testing methods, with a trend of increased cortical thickness in right TPOJ1 (2.16% in region of practical equivalence (ROPE)), and mostly inconclusive evidence of either differences or no differences for the remainder of the regions (ROPE between 2.5% and 97.5%). Using the test for practical equivalence based on the “HDI+ROPE decision rule” (41), we found that all six anatomical features remain undecided.

## Discussion

Surface-based analysis revealed that reduced adjusted volume and reduced cortical thickness in language-relevant regions are associated with the duration of restricted language experience in childhood. These effects occur primarily in inferior frontal and posterior temporal ROIs in the LH, as well as in superior temporal gyrus and inferior parietal ROIs in the RH, independent of language modality. These results indicate that childhood language experience plays a role in the development of the brain language system that can be observed years later in adulthood.

### Linking Adult Anatomical Measurements with Early Language Experience.

Human postnatal brain development is an intricate process involving interactions between genetic guidance and environmental experience. The relation between cortical thickness/volume and biological age is nonlinear, with an initial increase during the proliferation/synaptogenesis stage, followed by a decrease during the pruning stage (42) and a gradual decrease stabilizing at around 30 y (43). When biological age is held constant, findings to date suggest a strong relation between experience during childhood and brain growth, where reduced experience (as a result of environmental factors such as childhood poverty, low socio-economic status (SES), and neglect) has a negative impact on overall cortical growth, as reflected in reduced cortical thickness and volume, especially in frontal, temporal, and parietal regions (44–47). There also appears to be an extended period of cortical thickening in frontal and temporal regions between age 5 to 11 (48), suggesting prolonged neuroplasticity in these language-related regions when early experience could influence cortical growth. Cortical thickness and volume are also positively associated with language skills in young children (24, 49, 50) and in older adults (51).

Consistent with prior studies of children, the present study with adults found associations between the duration of restricted language access in childhood (as measured by AOA) and negative changes in cortical thickness/volume. As explained in the *Materials and Methods*, SES was not a factor in these present results.

### Left Fronto-Temporal Network.

For adjusted volume and cortical thickness measures in the LH, we found the effects of restricted childhood language in the inferior frontal (BA45) and the posterior middle temporal (TE1p) regions. Both regions are crucial for many aspects of language processing. Left BA45 is often considered to be crucial for language production, verbal working memory, as well as processing complex sentences in both spoken (52, 53) and sign languages (54). It is also sensitive to the amount of language experienced by young children (24, 55), and its development is associated with language performance at the sentence level (56). Left TE1p, located in the posterior middle temporal gyrus (pMTG), plays a major role in lexical access, again in both spoken (57, 58) and sign languages (59). It is also considered a hub for the language network, with extensive structural and functional connectivity with other language regions (60). In a recent model for sentence structure processing (61), these two regions are proposed to be two core components of the neuroanatomical network for syntax.

The present surface-based anatomical results parallel those of behavioral and BOLD signal results obtained in a previously reported fMRI study of the same participants (34). Grammatical judgment accuracy on the scanner task (see Table 2 d’GJ) was negatively associated with age onset of ASL, AOA (R = −0.65, t = −03.81, *P* < 0.001) and positively correlated with BOLD activation levels in the classic language areas of LH BA44 (Broca’s area) and BA22 (Wernicke’s area) and negatively correlated with BOLD activation in visual processing areas of BA 18/19 (lingual and middle occipital gyrus). The significant anatomical differences in the current study are in neighboring regions (BA45 and BA21), suggesting a relationship between anatomical development and functional activations as a result of reduced early language in the left fronto-temporal language network.

These anatomical findings help further explain the limited language outcomes observed among individuals who are born deaf and first experience accessible language long after infancy, especially at the morpho-syntactic levels of linguistic structure (for a review see ref. 33). The most affected regions (BA45 and pMTG) are often considered the core components of the sentence structure network (61). A previous study on the anatomical outcomes of severely reduced early language (62) also found reduced connectivity in arcuate fasciculus, a dorsal white matter fiber tract linking IFG and posterior temporal regions (63) that is crucial for language structure building. Previous studies of deaf late language signers have consistently found reduced neural activation in the left fronto-temporal language regions during processing of ASL signs (words) and sentences (34–36, 64). The anatomical correlates of restricted language experience we find here indicate that language experience during childhood contributes to the development of functional language processing in these crucial language regions. Without the anatomical foundations created by the interaction of brain growth and language experience, late first-language learners may lack sufficient neural resources to process linguistic structure to the same proficiency level as typical infant language learners.

A whole-brain voxel-based morphometry (VBM) analysis of the same data set from the present sample of deaf signers (37) found a negative correlation between age of ASL immersion and gray matter density in the occipital visual regions. Note that the effects of AOA on gray matter measures are the same in the present and previous studies, namely reduced gray matter with increased duration of restricted language experience. The prior study did not detect the differences in language-relevant regions, likely due to methodological constraints of the whole-brain VBM approach, as described in the *Methods*.

We did not find significant effects in the superior temporal gyrus and sulcus, the anterior temporal regions, and in the inferior parietal regions in the LH. These null effects suggest that early language experience has selective effects on the language network, with more pronounced effects found in the regions that are more often associated with structure building in language. In particular, the null effects in anterior temporal and inferior parietal language-relevant regions may relate to their sensitivity to non-linguistic, but meaning-bearing, environmental experience during early development. For example, the anterior temporal lobe is considered to be a semantic hub that processes domain-general semantic knowledge/memories (see ref. 65 for a review). Similarly, the inferior parietal regions are also known to be highly involved in domain-general social cognition (66). Non-linguistic semantic experience during early life, especially social interactions, might provide the necessary stimulation for relatively typical cortical development in these regions. This interpretation is consistent with previous studies showing rapid vocabulary development (67), an over-reliance on semantic information during sentence processing (27, 68), and relatively less affected white matter pathways linking anterior temporal and inferior frontal regions (62) in deaf individuals with restricted early language.

### RH Changes.

In addition to the significant effects in the classic left fronto-temporal language network, the results also show effects in some language areas in the RH. The effects observed in the RH are most significant in posterior superior temporal gyrus (pSTG) (A4, A5) and also in the ventral-anterior part of angular gyrus (TPOJ1).

RH pSTG has been reported to show cross-modal plasticity among congenitally deaf individuals, with responses to complex visual stimuli as well as motion (69–71). Given that ASL provides rich early visual stimuli with rapid and complex motion combinations, it is unsurprising that lacking such early visual experience, in addition to auditory experience, leads to reduced anatomical measures in this region.

RH TPOJ1, located in the ventral temporo-parieto-occipital junction, has previously been identified as a region showing less activation among hearing signers who learned ASL as a second language after childhood compared to hearing native signers (72). In addition, reduced selective activation for mental state in right temporo-parieto-occipital junction among deaf children with reduced early language has been found (73). Our current findings further suggest that early sign language experience may be crucial for the specialized differentiation in this region for sign language processing and/or language-constructed theory of mind.

Reduced volume and cortical thickness were also observed in inferior frontal and temporal regions (BA45, BA44, posterior STS, anterior MTG, see *SI Appendix*, Tables S1 and S2), although these effects were not as significant and did not survive the permutation correction. Children often show bilateral activation when processing language during the early years, while gradually shifting toward left lateralized activation over time (56, 74). Thus, it is possible that cortical growth of language regions in the RH is also potentially impacted by environmental language during early childhood.

### Modality Effects.

The results of the Bayesian analyses showed no evidence of significant differences or similarities between the deaf and hearing participants with an infant onset of language experience. Instead, the effects are associated with the deaf participants who experienced reduced language in childhood. These null results suggest that the experience-dependent effects in both hemispheres are associated with the developmental timing of language experience independent of sensory-motor modality. This is unsurprising, given extensive previous findings showing that the left fronto-temporal brain language network is shared by spoken and sign languages (75–78).

We did find a trend of increased cortical thickness in right TPOJ1 for the deaf native signers. As this region has been found to show reduced activation among hearing English speakers who learned ASL after childhood (72), it is possible that early sign language experience may contribute to this difference. Bayesian statistics indicate that we do not have enough evidence to conclude whether deaf and hearing individuals are similar or different in these brain regions. Given that we only examined ROIs with significant effects associated with highly restricted childhood language experience, and that the number of deaf signers with infant language input was small (N = 8), it is possible that there are anatomical differences related to language modality not detected here. However, language modality effects were not the focus of the present study. Other studies that explicitly compared deaf native signers, hearing native signers, and hearing sign-naïve speakers (79, 80) provide additional insights into this question.

The hearing individuals also showed relatively high variability in the anatomical measures, indicating that there may be unmeasured variables other than AOA that modulate the development of these brain areas. This is a remaining question that requires examination in future studies.

### Role of Early Language Experience on Cortical Development of Language Regions.

Together the results suggest selective effects of early language experience on the postnatal cortical development of language-relevant brain regions. First, the effects are specific to language regions. When compared to the somatomotor regions, the language regions show a greater reduction in volume and cortical thickness associated with the duration of restricted language experience in childhood. These effects are also selective within the language network and located primarily in the major hubs of this network (BA45 and posterior MTG) in the LH. These findings are consistent with previous studies showing functional and anatomical changes associated with the quantity and quality of language input among typically developing children (22–24). The results are further consistent with the functional and behavioral language profile of deaf late first-language learners who often struggle with aspects of language use (26–28). The effect sizes (adjusted R^2^ ranging from 0.367 to 0.469) and the percentage change in adjusted volume and cortical thickness (shown in Fig. 2) suggest that early language experience is associated with significant cortical growth in these language-related brain regions.

Because restricted early language also accompanies restricted social, conceptual, and potentially other types of experience that occur via language, it is difficult to disentangle the role of these nonverbal cognitive experience from language experience. However, individuals with more severe early social and conceptual deprivation, such as those from the Bucharest Early Intervention Project, who were raised from early infancy in institutions in Romania, showed much more widespread bilateral cortical thinning in frontal, temporal, and parietal regions (81). In contrast, the AOA effects in the current study are highly selective, both between language and non-language regions and within the left fronto-temporal and right homologous language network. One of the most affected regions in the LH (BA45) in the present study is also found to be sensitive to early language experience in children who hear (24). Both LH BA45 and pMTG are considered crucial language hubs and are highly involved in functions that are language specific. We did not find effects in regions within the language network that are more associated with conceptual or social functions, such as the ATL or the inferior parietal lobe (IPL) in the LH. This suggests that the effects observed in the LH are most likely due to reduced language experience.

As discussed above in the RH section, based on relevant literature, the effects in RH pSTG may reflect reduced cross-modal plasticity due to a lack of complex visual stimuli from early sign language input, a type of early experience that is sign language-driven but not limited to language experience. Also, the effects in RH TPOJ1 may potentially result from a lack of both language and language-constructed conceptual experience of mental states.

The current findings provide direct evidence that the human brain language system emerges as a product of both extended postnatal neuroplasticity in these regions sensitive to language and the young child’s environment. When the human brain matures in an environment with restricted language experience, the overall effect is reduced cortical growth in some crucial language-relevant brain areas. Decreased gray matter volume or thickness in left fronto-temporal language regions was linearly associated with the duration of restricted language access measured in number of years (up to 14 y), suggesting that the biological development of brain-language regions is sensitive to language experience throughout an extended sensitive period during early life. The bilateral effects also suggest that early language experience provides environmental stimulation promoting the growth of association cortices in both hemispheres. Given the profound effects of accessible language stimulation on cortical growth we find here, the present results indicate the need for a rich and accessible language environment for all children, including deaf children.

One remaining question is why adjusted volume and cortical thickness were more affected by AOA, while cortical area was less affected. More research mapping anatomical measurements with actual neurodevelopmental events is required to provide mechanistic explanations on this issue.

## Conclusion

Restricted language experience during early life in individuals born deaf is associated with negative changes in several anatomical features in bilateral language-relevant regions, especially in left fronto-temporal regions that are crucial for linguistic structure building. The effects are not observed when deaf native signers and hearing native speakers are directly compared, demonstrating the crucial role of early language experience on the development of the brain language system independent of language modality. Our findings show that development of the cortical layer of the brain language system is highly sensitive to childhood language experience.

## Materials and Methods

### Participants.

Data were analyzed from 43 adults. Twenty-two individuals were born severely or profoundly deaf and were the participants in previous studies (34, 37). Another 21 hearing adults served as controls and experienced spoken English from birth.

#### Deaf participants.

The deaf participants were recruited from among Deaf organizations in the Quebec and Ontario regions of Canada with a snowball method using the criteria of 1) being severely or profoundly deaf from birth, 2) using ASL as a primary language for 15 y or more, 3) having no documented other handicapping conditions or learning disabilities other than corrected vision, 4) being right-handed, and 5) being immersed in ASL from ages ranging from birth until 14. AOA was determined by the self-reported answer to the question, “How old were you when you first interacted daily with signers who were deaf.” This is a salient event in the lives of deaf signers, just as the age onset of language immersion is for hearing speakers and is the method used to determine AOA in studies of both signed and spoken language acquisition (82). To obtain a range of AOA, participants were recruited to represent three maturational epochs: infancy, 0 to 3 (native signers), early childhood from 4 to 8 y, and late childhood, 9 y and older. Although recruitment was based on these categories, AOA was used as a continuous variable in all the linear regression analyses reported here.

The recruitment yielded 22 deaf participants, 11 males, with a mean age of 38.6 (range 25 to 60, SD = 11.75). Five signers were immersed in ASL from birth by their deaf parents, two signers were immersed in ASL at the age of one and one signer at age 3 by their hearing parents, and 15 signers whose hearing parents did not sign were immersed in ASL between the ages of 4 and 14 in school settings with deaf peers (mean AOA = 5.77, SD = 4.95). Mean years of ASL, computed by subtracting age at testing from AOA, was 32.7 (range 18 to 53, SD = 11.03). Mean age of initial school entry was 4.1 y (range 3 to 7, SD = 1.54). One native signer was mainstreamed (attended public school with hearing peers); one attended an oral day school (speech actively encouraged and sign and gesture actively discouraged), and three attended day schools using Total Communication (TC, simultaneous sign and speech). Of the 15 non-native signers, 13 attended oral day schools where sign language and gesture were strictly prohibited as part of an educational strategy designed to promote speech in deaf children (83); two attended TC day schools. The performance of 18 participants on two nonverbal subtests of the WAIS (84) was within the normal range (Picture Completion: mean = 10.53, SD = 1.62; Picture Arrangement: mean = 12, SD = 3.0). None of these variables was related to AOA (Table 2).

Socioeconomic (SES) data were not collected from the participants who grew up in middle-class families. The language in the classroom for all the participants was English with the deaf participants also using ASL. If parental education level were used as a measure of SES, then the deaf participants from deaf families would have been from lower SES than those from hearing families^*^.

ASL proficiency was measured with two scanner tasks, Grammatical Judgement (GJ) and Phonemic Hand Judgement (PHJ). The stimuli consisted of mono-clausal, ASL sentences of two types, grammatical and ungrammatical counterparts, randomly presented. Participants indicated with a button press after each stimulus whether it was grammatical, yes/no, for the GJ task or whether the final sign of the stimulus was articulated with one hand, yes/no, for the PHJ task. The GJ task measures syntactic sensitivity, well known to correlate with AOA. The PHJ task measures phonemic sensitivity to single signs at the end of sentences, a more surface level task. To adjust for guessing, performance on both tasks was analyzed with D-prime, d′ = z(H)−z(F) where H is the proportion of yes responses to grammatical stimuli and F is the proportion of yes responses to ungrammatical stimuli. AOA predicted performance on the scanner GJ task (R = −0.65, *P* < 0.001) but not on the PHJ task (R = −0.29, *P* = 0.19, ref. 34).

The BOLD signal change results from the same participants (34) suggest cortical sensitivity to language experience during childhood in several left frontal-temporal language regions, including IFG (BA44) and superior temporal gyrus (BA22). These behavioral and functional neural activation results are consistent with other studies on deaf signers with delayed sign language onset (35, 36, 64), showing atypical language performance especially at the morpho-syntactic level and reduced neural activation in the typical language regions with little functional compensatory mechanisms.

#### Hearing participants.

A control group of right-handed hearing subjects matched for age and gender who had never been exposed to sign language (n = 21, 7 males, 14 females, average age = 30.95 y, SD = 5.35) was included in this study. These subjects had been previously scanned at the MNI using the same scanner and imaging parameters. All studies related to participants were approved by the MNI Research Ethics Board. Hearing control participants were approached by their initial study PI and asked if they approved their MRI being used for secondary use and provided email responses releasing their MRI for use. They were all English speakers, but we do not have additional information about their language background, as all the images were anonymized. The hearing participants were recruited from the Quebec area, therefore experienced a dual French and English bilingual environment. Any bilingual-related anatomical changes should be similar across both deaf and hearing native groups. Although no SES data were collected, they would be characterized as middle class.

### Imaging Acquisition.

All participants were scanned at the MNI using a 1.5T Siemens Sonata imager. T1 weighted magnetic resonance (MR) images was acquired using 3D fast low-angle shot (FLASH) (TR = 22 ms, TE = 9.2 ms, flip angle = 30°, matrix size = 256 × 256, number of slices = 170, voxel size = 1 × 1 × 1 mm^3^).

### Preprocessing and Cortical Reconstruction.

One of the limitations of the VBM analysis is that the voxel registration can be distorted for aligning variable cortical regions for group comparisons (85, 86). Higher-order cortical regions, including crucial language-relevant areas, often show more variability, and the cortical folding patterns can be used to better map these regions across individuals (87, 88). Therefore, surface-based analysis using cortical folding patterns (38) can provide a more accurate mapping of the brain ROI. Also, with the recent advancement in human brain mapping, better parcellation and brain atlases are now available for ROI-based analyses of language-relevant regions.

We used the recon-all function in FreeSurfer (38) to process the structural MRI images and to perform cortical reconstruction. The processing steps included motion and intensity correction, skull stripping, common reference registration, and volume segmentation. Cortical surface reconstruction was performed in the native anatomical space for each individual. Three surface-based morphological measurements were obtained from the segmentation output: gray matter volume, cortical thickness, and cortical surface area. Detailed description of the surface morphometry methods and how these anatomical features are derived can be found in ref. 38. We calculated adjusted volume for each ROI by taking the raw volume from the respective ROI and adjusting it using the individual’s estimated Total Intracranial Volume (eTIV, also known as ICV), also derived from the recon-all function in FreeSurfer. We examined the correlation between potential artifacts (mean motion, eTIV) and the target predictor, age of ASL onset (AOA). No significant correlation was found between AOA and mean motion (r = 0.08, *P* = 0.69), and between AOA and eTIV (r = −0.16, *P* = 0.46). The adjusted volume value represents the basis point (0.01%) of the eTIV.

### Parcellation.

After reconstructing the cortical surface for each individual, we used a multi-modal cortical parcellation map to identify target language regions. The parcellation map was developed as part of the Human Connectome Project (HCP). It includes 180 areas in each hemisphere. The parcellation was determined by changes in cortical architecture, function, connectivity, and/or topography, using multimodal imaging from 210 healthy young adults (39). We used customized MATLAB scripts to project the HCP cortical parcels from Workbench (89) 32k to the FreeSurfer (38) template following the instructions from (90).

### Language ROIs.

**Fig. 4.**
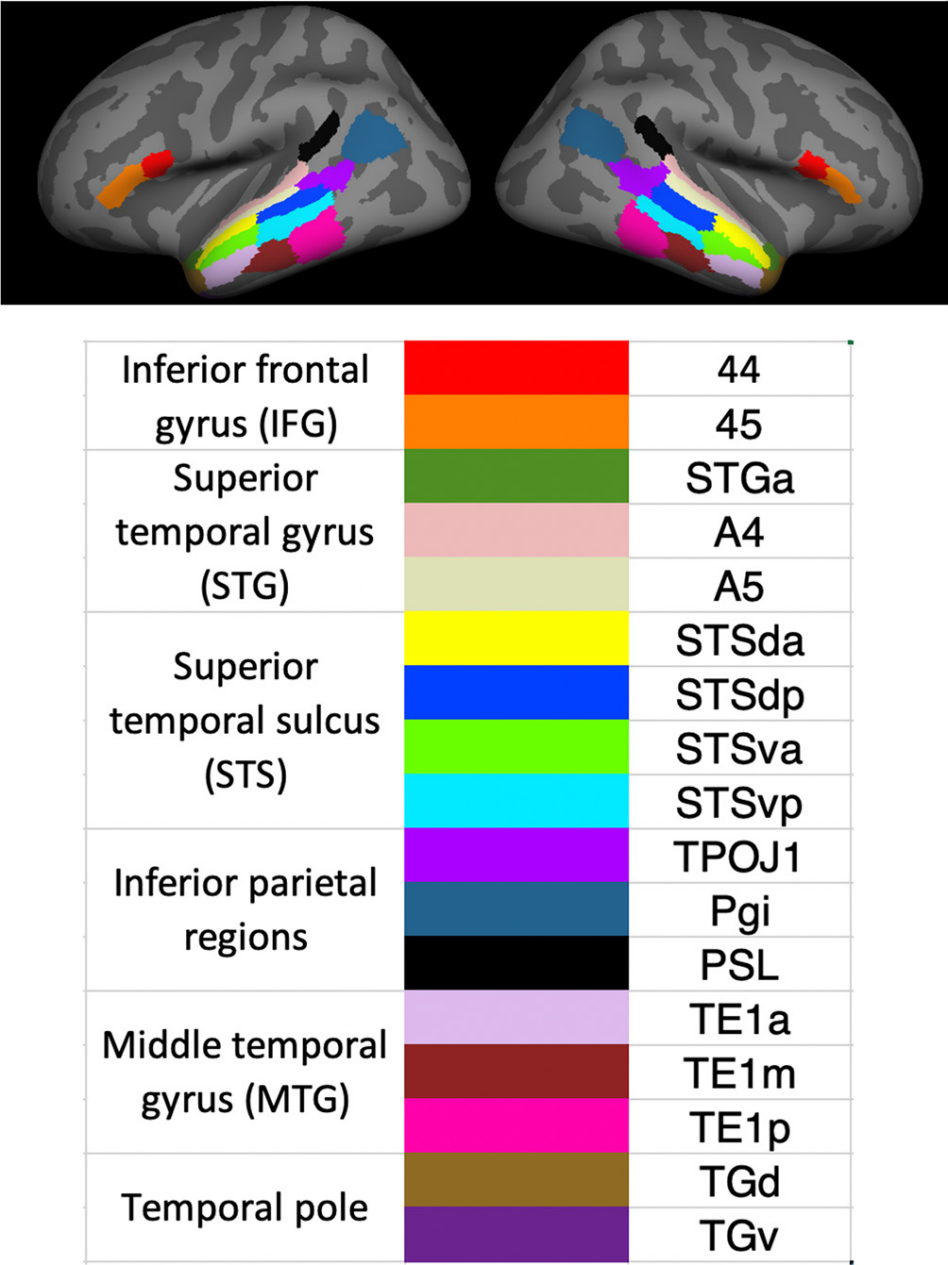
Color-coded bilateral language ROIs selected from the HCP cortical parcellation map, shown on the FreeSurfer surface template.

We choose 17 language-relevant ROIs from the HCP atlas based on previous neurolinguistics literature (91) as well as the connectivity and functional activation patterns as reported in the HCP paper: inferior frontal regions (BA44, BA45); superior temporal regions (STGa, A4, A5, STSda, STSdp, STSva, STSvp); anterior temporal regions (TGd, TGv); middle temporal regions (TE1a, TE1m, TE1p); and inferior parietal regions (PGi, TPOJ1, PSL). Previous studies on sign language neural processing suggest that these language-relevant regions are also activated during sign language processing (75, 77, 92, 93). Given recent findings on bilateral activation during early years of language processing (74), we also included the homologous ROIs in the RH. Fig. 4 shows the color-coded ROIs on the Freesurfer surface template. Detailed descriptions on where these ROIs are located and how the boundaries are determined in the HCP atlas can be found in the supplementary materials of Glasser et al. (39).

### Somatomotor ROIs as Controls.

Somatomotor regions were selected as controls in the current study, given their relatively early maturation (7–9), and largely non-linguistic functions, indicating less influence from early language experience. We choose 19 somatomotor ROIs from the HCP atlas based on ref. 94: 4, 3b, 5m, 5L, 24dd, 24dv, 7AL, 7PC, 1, 2, 3a, 6d, 6mp, 6v, OP4, OP1, OP2-3, FOP2, lg.

### ROI-Based Analyses.

We analyzed the anatomical data in three steps. First, for each of the anatomical features, we aggregated the values across all language-relevant ROIs (sum of adjusted volume, average of thickness, sum of cortical area), and fitted linear models using AOA and hemisphere and their interaction as the target variables, and gender and age as covariates, using the lme4 package in R (95) and the lm function. Second, we analyzed the role of AOA among deaf individuals in each of the selected language ROIs. For each anatomical measurement, we fitted multivariate linear models using AOA as the main independent variable, and the corresponding values from the language-relevant ROIs as dependent variables using the lme4 package in R (95) and the lm with cbind function. We used the max-t method permutation to correct for the p values given correlated multiple comparisons (34 comparisons total for each measurement), using the coin package (96) and the independence_test function, with 100,000 resamples. Finally, for those anatomical features showing significant AOA effects, we compared the deaf native group with the hearing non-signer group to see whether the effects are associated with early ASL exposure only, or rather, come from early language exposure regardless of modality. We examined the homogeneity of variance across these two groups using the Levene’s test. For each ROI/measure combination with significant AOA effects, we fitted linear models with group (hearing vs. deaf native) as a main factor and with age and gender as covariates, using the lme4 package in R (95) and the lm function. The Bayesian models were fit using the rstan package (97) and the stan_glm function. The practical equivalence tests were conducted using the bayestestR package (98) and the equivalence_test function.

## Supplementary Material

Appendix 01 (PDF)

## Data Availability

All analysis codes and anatomical measurements data used in this study are available at https://doi.org/10.5281/zenodo.7402337 (99).

## Footnotes

^*^Survey data indicate 52% deaf adults in the USA as having some college with a 25.5% pay gap compared to hearing adults with comparable education levels (85).
