# Supplementary material for: Restricted language access during childhood affects adult brain structure in selective language regions
Source: Proc Natl Acad Sci U S A. 2023 Feb 6;120(7):e2215423120. doi: 10.1073/pnas.2215423120 (PMC9963327; doi:10.1073/pnas.2215423120)
Supplement: Supplementary file 1 — Appendix 01 (PDF) [file pnas.2215423120.sapp.pdf]

Supplemental Information
Table S1-3 shows the mean values and standard deviations of each group.

Table S1: Adjusted volume

	Left hemisphere				Right hemisphere			
**ROI**	Deaf Native (N=8)	Deaf Early (N=8)	Deaf Late (N=6)	Hearing (N=21)	Deaf Native (N=8)	Deaf Early (N=8)	Deaf Late (N=6)	Hearing (N=21)
**44**	9.91 (1.77)	8.65 (1.97)	8.79 (1.03)	9.35 (1.20)	11.06 (2.26)	9.34 (0.76)	9.13 (1.89)	11.47 (1.41)
**45**	13.91 (1.63)	11.48 (2.33)	10.57 (1.06)	13.79 (2.65)	9.11 (1.53)	7.17 (1.77)	6.61 (0.84)	8.76 (1.53)
**STGa**	7.72 (1.17)	6.68 (1.47)	7.14 (1.12)	8.16 (1.60)	6.25 (1.15)	5.37 (1.53)	5.78 (0.47)	7.02 (1.21)
**A4**	11.05 (0.77)	10.36 (2.73)	9.54 (0.83)	11.00 (1.94)	10.71 (0.68)	9.05 (1.64)	8.61 (1.36)	10.63 (1.67)
**A5**	9.42 (1.18)	9.30 (1.82)	9.07 (1.03)	9.23 (2.07)	12.38 (0.90)	10.46 (1.39)	10.21 (1.69)	13.02 (1.86)
**STSda**	7.10 (0.69)	7.70 (2.98)	7.87 (2.12)	7.18 (2.23)	8.56 (1.57)	7.55 (2.52)	8.11 (1.36)	10.18 (0.76)
**STSdp**	5.85 (1.10)	5.87 (1.01)	5.72 (1.02)	6.37 (0.81)	7.52 (1.23)	5.96 (0.79)	6.32 (1.48)	7.48 (0.72)
**STSva**	6.22 (1.01)	5.90 (1.22)	5.91 (1.60)	6.44 (1.78)	7.75 (0.84)	6.66 (1.10)	6.76 (1.82)	8.32 (1.04)
**STSvp**	9.22 (1.54)	7.74 (1.78)	8.40 (1.45)	9.41 (1.92)	9.38 (1.53)	7.96 (1.43)	9.03 (1.67)	9.81 (1.55)
**TGd**	38.60 (3.54)	37.38 (5.40)	36.17 (5.07)	39.93 (4.46)	33.14 (3.15)	34.82 (5.17)	38.74 (3.92)	36.99 (3.59)

**TGv**	14.70 (1.91)	15.00 (3.19)	13.89 (2.39)	15.04 (3.12)	14.75 (1.87)	15.31 (2.73)	15.38 (4.07)	16.69 (3.01)
**TE1a**	17.41 (1.95)	14.97 (2.32)	16.38 (2.64)	17.16 (2.40)	12.95 (1.31)	13.06 (2.75)	12.96 (2.39)	14.34 (1.91)
**TE1m**	12.21 (1.82)	8.46 (1.73)	10.37 (1.10)	11.49 (1.18)	9.40 (2.24)	8.72 (1.71)	9.09 (2.20)	9.94 (1.25)
**TE1p**	21.43 (3.03)	17.20 (4.19)	16.90 (2.42)	20.70 (3.33)	19.65 (1.95)	17.81 (2.87)	17.54 (2.93)	18.59 (1.41)
**PGi**	15.60 (2.90)	13.68 (3.16)	14.66 (2,84)	15.21 (2.36)	17.39 (3.80)	18.49 (7.35)	18.73 (4.65)	20.21 (3.92)
**TPOJ1**	6.27 (1.44)	5.38 (0.74)	5.77 (1.10)	6.83 (1.76)	12.35 (1.94)	10.53 (1.75)	11.42 (2.09)	12.43 (2.37)
**PSL**	7.82 (1.68)	7.84 (1.74)	6.21 (1.05)	7.72 (2.03)	9.06 (1.59)	7.37 (0.90)	8.06 (0.75)	9.61 (1.06)

Table S2: Cortical thickness

	**Left hemisphere**				**Right hemisphere**			
**ROI**	Deaf Native (N=8)	Deaf Early (N=8)	Deaf Late (N=6)	Hearing (N=21)	Deaf Native (N=8)	Deaf Early (N=8)	Deaf Late (N=6)	Hearing (N=21)
**44**	2.83 (0.24)	2.72 (0.24)	2.62 (0.19)	2.80 (0.21)	2.77 (0.22)	2.77 (0.25)	2.64 (0.27)	2.97 (0.29)
**45**	2.67 (0.15)	2.55 (0.18)	2.52 (0.16)	2.76 (0.16)	2.80 (0.15)	2.61 (0.12)	2.51 (0.22)	2.81 (0.22)
**STGa**	3.75 (0.36)	3.44 (0.53)	3.67 (0.12)	3.61 (0.48)	3.59 (0.38)	3.36 (0.58)	3.53 (0.27)	3.59 (0.37)

**A4**	3.22 (0.15)	3.01 (0.22)	2.99 (0.19)	3.03 (0.22)	3.26 (0.18)	3.15 (0.29)	2.97 (0.15)	3.13 (0.25)
**A5**	3.14 (0.12)	3.04 (0.22)	2.95 (0.20)	3.03 (0.17)	3.20 (0.12)	2.98 (0.14)	2.90 (0.14)	3.07 (0.18)
**STSda**	2.87 (0.18)	2.74 (0.29)	2.69 (0.15)	2.65 (0.18)	2.79 (0.15)	2.70 (0.18)	2.63 (0.17)	2.79 (0.17)
**STSdp**	2.57 (0.11)	2.47 (0.18)	2.41 (0.09)	2.51 (0.16)	2.67 (0.11)	2.47 (0.13)	2.42 (0.20)	2.55 (0.16)
**STSva**	2.64 (0.18)	2.51 (0.24)	2.47 (0.18)	2.62 (0.24)	2.72 (0.14)	2.72 (0.25)	2.59 (0.19)	2.69 (0.17)
**STSvp**	2.67 (0.11)	2.53 (0.23)	2.47 (0.10)	2.70 (0.22)	2.96 (0.15)	2.91 (0.16)	2.74 (0.20)	2.88 (0.20)
**TGd**	3.56 (0.25)	3.42 (0.29)	3.36 (0.16)	3.43 (0.36)	3.38 (0.27)	3.37 (0.28)	3.42 (0.26)	3.40 (0.27)
**TGv**	3.23 (0.34)	3.19 (0.38)	2.93 (0.18)	3.10 (0.48)	3.19 (0.43)	3.14 (0.36)	2.92 (0.35)	3.24 (0.33)
**TE1a**	3.33 (0.22)	3.17 (0.32)	3.16 (0.15)	3.19 (0.19)	3.50 (0.17)	3.36 (0.25)	3.27 (0.35)	3.35 (0.41)
**TE1m**	3.01 (0.22)	2.76 (0.30)	2.75 (0.31)	2.93 (0.18)	3.12 (0.27)	2.85 (0.41)	2.77 (0.19)	2.94 (0.34)
**TE1p**	3.07 (0.06)	2.85 (0.21)	2.78 (0.19)	2.94 (0.20)	3.05 (0.16)	2.79 (0.23)	2.88 (0.10)	2.94 (0.23)
**PGi**	2.53 (0.10)	2.36 (0.21)	2.49 (0.14)	2.49 (0.12)	2.54 (0.09)	2.35 (0.16)	2.46 (0.14)	2.52 (0.17)
**TPOJ1**	2.63 (0.16)	2.46 (0.20)	2.47 (0.12)	2.60 (0.19)	2.75 (0.12)	2.53 (0.17)	2.49 (0.11)	2.61 (0.20)

PSL	2.77 (0.29)	2.67 (0.29)	2.58 (0.16)	2.77 (0.20)	2.96 (0.25)	2.73 (0.15)	2.69 (0.18)	2.88 (0.12)

Table S3: Cortical area

	**Left hemisphere**				**Right hemisphere**			
**ROI**	Deaf Native (N=8)	Deaf Early (N=8)	Deaf Late (N=6)	Hearing (N=21)	Deaf Native (N=8)	Deaf Early (N=8)	Deaf Late (N=6)	Hearing (N=21)
**44**	465.75 (72.40)	434.38 (59.36)	417.00 (45.44)	404.76 (61.02)	510.25 (90.06)	440.13 (46.92)	448.33 (106.33)	452.14 (54.46)
**45**	686.38 (93.61)	622.88 (108.72)	556.33 (72.61)	609.95 (105.65)	429.25 (91.94)	388.13 (91.73)	347.00 (76.94)	386.33 (80.77)
**STGa**	206.88 (22.50)	219.63 (53.75)	202.67 (30.69)	215.29 (25.71)	190.00 (38.22)	187.75 (22.88)	178.17 (18.65)	197.90 (17.63)
**A4**	368.75 (47.39)	411.13 (94.57)	341.00 (45.87)	368.75 (56.78)	378.24 (51.95)	344.50 (61.95)	317.67 (49.94)	363.43 (37.80)
**A5**	367.00 (54.56)	379.88 (73.71)	372.33 (61.86)	351.43 (57.15)	499.38 (56.24)	478.00 (87.03)	453.83 (81.83)	534.67 (47.46)
**STSda**	407.25 (43.88)	441.50 (100.87)	437.33 (58.15)	387.19 (48.92)	497.50 (61.50)	475.50 (110.93)	484.50 (100.60)	553.90 (40.70)
**STSdp**	420.38 (61.00)	428.13 (70.80)	410.83 (54.33)	429.62 (82.08)	527.88 (62.64)	487.88 (64.87)	474.17 (76.24)	514.67 (53.02)

**STSva**	449.63 (71.28)	442.88 (81.43)	447.83 (87.06)	413.90 (73.21)	440.25 (54.72)	392.63 (74.42)	404.67 (106.90)	451.43 (40.33)
**STSvp**	557.00 (71.77)	511.38 (109.45)	531.17 (79.96)	528.67 (133.82)	452.13 (80.75)	409.75 (67.41)	456.00 (70.69)	457.05 (73.85)
**TGd**	1279.63 (137.29)	1344.38 (136.77)	1285.33 (173.05)	1263.52 (110.04)	1126.38 (116.47)	1241.13 (119.11)	1270.33 (142.21)	1161.14 (70.61)
**TGv**	497.88 (67.86)	521.13 (123.62)	504.67 (62.78)	492.90 (52.43)	502.63 (61.74)	562.63 (67.31)	552.17 (66.23)	503.24 (51.88)
**TE1a**	586.50 (98.29)	552.25 (77.25)	577.50 (120.97)	565.62 (59.82)	431.25 (66.86)	452.63 (80.00)	462.00 (84.34)	456.43 (32.69)
**TE1m**	523.63 (68.44)	436.00 (99.75)	500.00 (95.18)	494.43 (76.82)	408.88 (98.89)	423.50 (72.47)	440.33 (103.31)	430.71 (68.88)
**TE1p**	916.75 (156.73)	825.50 (212.46)	794.83 (136.04)	839.52 (138.99)	843.88 (79.10)	877.13 (131.95)	799.50 (171.95)	790.24 (113.37)
**PGi**	989.88 (174.65)	977.50 (214.17)	929.33 (206.63)	911.38 (150.08)	1057.75 (207.93)	1266.88 (479.56)	1078.33 (142.38)	1110.57 (224.75)
**TPOJ1**	396.38 (75.43)	380.75 (61.28)	385.83 (83.73)	401.86 (57.44)	719.25 (137.00)	697.50 (121.94)	698.17 (52.85)	744.52 (106.43)
**PSL**	398.38 (63.47)	443.75 (107.70)	345.67 (62.06)	368.48 (89.58)	415.13 (72.82)	380.88 (56.89)	400.67 (113.92)	406.81 (71.50)
